# Supplementary material for: ChAdOx1 nCoV-19 (AZD1222) protects Syrian hamsters against SARS-CoV-2 B.1.351 and B.1.1.7
Source: Nat Commun. 2021 Oct 7;12:5868. doi: 10.1038/s41467-021-26178-y (PMC8497486; doi:10.1038/s41467-021-26178-y)
Supplement: Supplementary file 1 — Supplementary Information [file 41467_2021_26178_MOESM1_ESM.pdf]

Table S1. Primers and probes used. NA = not applicable.

| Primer name | Sequence | Catalogue number IDT |
|---|---|---|
| E_Sarbeco_F1 Forward | ACAGGTACGTTAATAGTTAATAGCGT | 10006889 |
| E_Sarbeco_R2 Reverse | ATATTGCAGCAGTACGCACACA | 10006891 |
| E_Sarbeco_P1 Probe | ACACTAGCCATCCTTACTGCGCTTCG | 10006893 |
| sgLeadSARSCoV2-F | CGATCTCTTGTAGATCTGTTCTC | NA |
